# “Expecting the Unexpected:” Nurses' Response and Preparedness of Terrorism-Related Disaster Events in Quetta City, Pakistan

**DOI:** 10.3389/fpubh.2021.695143

**Published:** 2021-05-25

**Authors:** Fazal ur Rehman Khilji, Abdul Raziq, Maryam Shoaib, Nosheen Sikander Baloch, Shanaz Raza, Zaffar Iqbal, Rabia Ishaq, Sajjad Haider, Qaiser Iqbal, Nafees Ahmad, Fahad Saleem

**Affiliations:** ^1^Faculty of Pharmacy and Health Sciences, University of Balochistan Quetta, Quetta, Pakistan; ^2^Department of Statistics, University of Balochistan Quetta, Quetta, Pakistan; ^3^Sandeman Provincial Hospital, Quetta, Pakistan; ^4^Department of Gynecology and Obstetrics, Bolan Medical College, Quetta, Pakistan; ^5^Department of Pharmacy, Sardar Bahadur Khan Womens' University, Quetta, Pakistan; ^6^Health Department, Government of Balochistan, Quetta, Pakistan

**Keywords:** medication services, terrorism, disaster, preparedness and response, nurses

## Abstract

**Background:** In addition to the psychiatric and societal misery, terrorism places an exceptional burden while delivering healthcare services. Accordingly, a responsive and well-prepared healthcare system ensures effective management of terrorism-related events. Within this context, with a strong historic grounding in addressing situations of societal crisis nurses are well-placed in contributing to the global arena of humanitarian policy and social research. Therefore, assessing their response and preparedness is vital in effective management of a terrorism-related disaster. For that very reason, we aimed to evaluate nurses' preparedness and response toward terrorism-related disaster events in Quetta city, Pakistan.

**Methods:** A qualitative design was adopted to explore nurses' response and preparedness of terrorism-related disaster events. By using a semi-structured interview guide through the phenomenology-based approach, in-depth, face-to-face interviews were conducted. Nurses practicing at the Trauma Center of Sandeman Provincial Hospital (SPH), Quetta, were approached for the study. All interviews were audio-taped, transcribed verbatim, and were then analyzed for thematic contents by the standard content analysis framework.

**Results:** Fifteen nurses were interviewed and thematic content analysis revealed five themes. All nurses have experienced, responded to, and managed terrorism-related disaster events. They were prepared both professionally and psychologically in dealing with a terrorism-related disaster. Among limitations, space and workforce were highlighted by almost all the respondents. Lack of disaster-related curricula, absence of a protocol, recurrence of the disaster, and hostile behavior of victim's attendants during an emergency were highlighted as a key barrier toward terrorism-related disaster management.

**Conclusion:** The skills and expertise needed to address a terrorism-related disaster are well-understood by the nurses but are lacking for various reasons. In addition to the review and adaption of the nursing curriculum specifically for terrorism-related disaster management, collaboration and dialogue between various stakeholders is required to efficiently manage terrorism-related disaster events.

## Introduction

Defined as “*an occurrence disrupting the normal conditions of existence and causing a level of suffering that exceeds the capacity of adjustment of the affected community*” (1), disasters are a complex global problem (2). Throughout history, disasters have produced adversative consequences (3) and literature reveals that the prevalence of disasters has risen during the past decades (4). Disasters vary from localized events to large-scale urgencies that cause mass casualties with devastating results and adversely influence the health of a population (5).

Parallel to the natural disasters and emergencies, terrorism-related or man-made disasters have fueled since the 1970s (6). Arise in terrorist-related events throughout the previous decades is reported (4). Although the Global Terrorism Index (7) described a decline of terrorism-related events in recent years, 15,952 global deaths were still reported in 2019. Furthermore, the number of countries affected by terrorism remained high as 71 countries recorded at least one death from terrorism in 2018, the second highest number of countries since 2002 (7). In term of regions, South Asia has had the highest impact from terrorism since 2002, while Central America and the Caribbean region have had the lowest impact. Lastly, the global economic impact of terrorism in 2018 was US $33 billion in constant purchasing power parity, a decline of 38 percent from its 2017 level (7). It should be remembered that the figure is conservative and does not include indirect impacts on business, investment and the costs associated with security agencies in countering terrorism. In addition, there are wide-ranging economic consequences that have the potential to spread quickly through the global economy with significant social ramifications.

Terrorism-related disasters not only cause catastrophic destruction of life and public infrastructures, but also, disrupt normal healthcare delivery and, to a great extent, the ability to cope at all levels with disaster victims. Becker and Middleton highlighted that along with first responders, healthcare institutes and professionals play a central role in addressing the health impacts of terrorism-related events (8). They provide immediate and critical care during emergencies, respond to disasters and preserve the safety of the community (9, 10). As terrorism-related disasters creates chaos and muddle in the society, an effective disaster management plan is needed that can help to alleviate some of the pandemonium wrought by the unexpected event. Correlating terrorism-related events and healthcare system, it is critical that the institutes and professionals are professionally prepared and trained to manage the unfortunate events. Accordingly, disaster management and preparedness of the healthcare system is widely studied in literature (11–13). Since the September 11 terrorist attacks, government agencies and professional societies around the globe have focused greater attention on the importance of healthcare professionals' preparedness and response toward terrorism-related events (14). Within this context, although the Institute of Medicine (IMS) reported improved preparedness and response of emergency departments toward terrorist-related event in the USA, major hurdles and challenges were also highlighted (15). On the contrary, information about the response and preparedness toward a terrorism-related disaster is scarce from the developing world but based on the developed world example (15), we can hypothesize that it is far below satisfaction.

Shifting our concerns to terrorism-related events in a developing country like Pakistan, the country was identified as one of the seriously high overwhelmed states being affected by terrorism-related disasters (16, 17). From 2002 to 2009, Pakistan was considered viable of a 12 percent rise in terrorism across the globe (16). Concerning the Pakistan Institute of Peace Studies (PIPS) security description, the terrorist violence resulted in 19,165 killings of individuals during the years 2003 and 2009 including civilians, officials of law enforcement agencies as well as the terrorists. Additionally, countrywide 2,113 (highest among all) terrorist attacks were acknowledged in the year 2010, which resulted in 2,913 deaths of individuals, and additionally 5,824 were wounded (18). In addition to the economical and societal obliteration, the terrorism-related events always place an additional burden on the already weakened healthcare system of the country. With deprived infrastructure, constraint of human resources and financial limitations (19, 20), healthcare system of Pakistan face additional burden once a terrorism-related event occurs. Under such conditions, it is vital that healthcare institutes and professionals are prepared and trained to manage a terrorism-related event under limited resources and financial constraints otherwise the results can be devastative. Also, understanding the preparedness and management is important in order to effectively handle the disaster in future. However, to the best of our knowledge and through extensive literature review, the information on healthcare professional's preparedness and response to a terrorism-related disaster in Pakistan is not reported in literature. Considering the high incidence of terrorism-related events in Pakistan and scarcity of information on its management, we designed this study to evaluate the response and preparedness of healthcare professionals (nurses) toward terrorism-related events in Quetta city, Pakistan.

## Methods

### Study Design and Settings

We adopted a qualitative study design (in-depth, face-to-face interviews). This method is flexible and consents to detailed exploration of respondents' attitudes, experiences, and intentions (21, 22). Also, qualitative studies generate a wide range of ideas and opinions that individuals carry about issues, as well as divulge viewpoint and differences among groups (23, 24). But most importantly, qualitative methods fill the gaps that are left unexposed by survey-based research specifically when it comes to under-discovered research areas (25). Therefore, inline to the objectives of this study, a qualitative design was a unmatched choice for inductive approaches aimed at generating concepts and hypothesis which have far more potential for research than any other models (26).

The study site was the Trauma Center Quetta (TC). Established in 2016 and located within the premises of Sandeman Provincial Hospital (SPH) Quetta, the TC is well-equipped and deals with emergent situations and provides prompt health care facilities 24/7 to the victims of terrorism-related as well as general trauma events (27). Prior to the establishment of TC, the causality department of SPH managed emergencies including the victims of terrorism. The TC is a 30-bedded facility with 24 physicians, 33 nurses, and 7 pharmacists stationed to offer healthcare services.

### Study Participants, Criteria, and Sampling

Registered nurses with minimum nursing diploma, stationed and practicing at the TC and consenting to participate in the study were approached for data collection. Based on our objective (nurses involved in managing terrorism-related disaster event), it was apparent to adopt the purposive sampling method (28). Nurses on rotations, stationed in the TC as part-timers and not willing to participate were excluded.

### The Interview Guide (Validation, Reliability, and Pilot Study)

The research team constructed a semi-structured interview guide after an extensive literature review (29–33), through expert panel discussion, and experience sharing (34–36). The guide was established with widely framed, open-ended questions that gave enough opportunities to the respondents. Parallel, nurses were also encouraged to provide their own narratives and to share further information relevant to terrorism-related disaster management.

The guide was constructed in the English language and was translated into Urdu (National language of Pakistan) by an independent linguistic expert. The translated guide was back-translated into English to avoid discrepancies by another independent translator (37). With little amendments in translation, the guide was subjected to face and content validity through a panel of experts (senior nurses and physicians) having experience in terrorism-related disaster management. Once the validity was ensured, the guide was piloted with four nurses to ensure that topics to be discussed were at the level that respondents would comprehend with ease. The preliminary data and conclusion confirmed that the discussion topics were enough and appropriately phrased to answer research questions and to minimize validity and reliability threats. As the validity and reliability of the discussion guide was ensured, it was made available for the main study. Data and participants of the pilot study were not included in the final analysis.

We used the triangulation and member checks to establish credibility that contributed to trustworthiness. The data was audited to determine if the research situation applies to the similar circumstances. It was made sure that analysis conducted in a precise, consistent, and exhaustive manner through recording, systematizing, and disclosing the methods of analysis with enough detail to ensure that process is credible.

### Interview Procedure, Data Collection, and Analysis

The first author conducted the interviews at the TC. The interviewer carries a Masters degree in practice research and was professionally trained and ensured before the interviews were conducted. Keeping the nature of the study and the ease of the respondents into consideration, the interviews were conducted in Urdu. All participants were briefed about the study objectives before the interviews. A debriefing session was again conducted at the end of the discussion. The interviews started with an ice-breaking session. Probing questions were asked in between conversations to clarify the meanings of responses and to gain insight of the topic being discussed.

Each interview was audio-recorded that lasted for ~45 min to 1 h. To draw in-depth views, the freedom to express additional reviews and comments was given to the nurses. The second author acted as an observer while the third author assisted in monitoring the field notes, facial expressions and body language that complemented the audio recordings. Interviews were conducted until thematic saturation was reached and no new information was discovered. This redundancy signals to researchers that data collection may cease (38, 39). The interviews were conducted and transcribed the next day in order to ensure that saturation goes parallel. The research team analyzed the recordings (verbatim) and later arranged an informal gathering where nurses were presented with the finalized interview scripts (40). They were asked for confirmation of precision and accuracy of words, ideas, and jargon used during the script analysis. Once confirmed, the transcripts were translated into English by another independent translator for thematic content analysis (41, 42). NVivo® was used for coding and analysis through iterations (43) and inconsistencies were resolved through mutual consensus. All emerging themes and subthemes were discussed among the research team for accuracy and were presented for data inference and interpretation.

### Ethical Approval

Institutional review board at the Faculty of Pharmacy and Health Sciences, University of Baluchistan approved the study protocol (UoB/Reg:/GSO/67). Written consent was taken from the respondents before the interviews. The nurses were introduced to the nature of the research prior to the beginning of the interviews, were made secure of the confidentiality of their responses and their right to withdraw from the study.

## Results

### Demographic Data

Fifteen nurses took part in the interview process out of which 9 (60%) were females. All respondents were practicing as a staff nurse and had a diploma in nursing. Thirteen (86.7%) respondents had an overall nursing experience of fewer than 10 years (median = 8). While talking specifically about working at the TC, 8 (53.3%) had an experience of more than 18 months. None of the respondents was trained precisely in terms of managing disasters as shown in Table 1.

**Table 1:** Demographic characteristics of the respondents.

**Characteristics**	**Frequency**	**Percentage**
**Age (years)**		
18-27	7	46.7
28-37	8	53.3
**Gender**		
Male	6	40.0
Female	9	60.0
**Education**		
Nursing diploma	15	100
**Nursing experience (years)**		
1-10	13	86.7
>10	2	13.3
**Experience at Trauma center (months)**		
6-12	5	33.3
13-18	2	13.3
>18	8	53.3
**Current position**		
Staff nurse	15	100
**Specialization**		
None	12	80.0
Cardiac	2	13.3
Psychiatry	1	6.6
**Specialization in Disaster Management**		
None	15	100

Thematic content analysis resulted in five major themes (Table 2). The themes and sub-themes are discussed as under.

**Table 2:** Schematic presentation of themes and sub themes identified during data analysis.

**Theme 1: Terrorism-related disaster event (experience, information source and call-up mechanism)**		
**Sub theme 1(a):**		**Sub theme 1(b):**
Experience of terrorism-related disaster event(s)		Disaster-related information sources and call-up mechanism
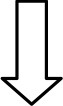		
**Theme 2: Response toward the terrorism-based disaster event**		
**Sub theme 2(a):**	**Sub theme 2(b):**	**Sub theme 2(c):**
Professional response	Personal response	Inclusive response
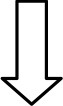		
**Theme 3: Preparedness of terrorism-related disaster management**		
**Sub theme 3(a):**	**Sub theme 3(b):**	**Sub theme 3(c):**
Current level of knowledge and familiarity of terrorism-related disaster management	Workforce, infrastructure, and supplies	Triage, communication and coordination
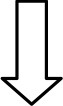		
**Theme 4: Barriers toward terrorism-related disaster management**		
**Sub theme 4(a):**	**Sub theme 4(b):**	**Sub theme 4(c):**
Safety concerns and issues	Lack of disaster management content	Lack of drills and hands-on trainings
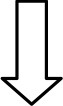		
**Theme 5: Suggestions and recommendations**		

### Theme 1: Terrorism-Related Disaster Event (Experience, Information Source, and Call-Up Mechanism)

#### Subtheme 1a: Experience of Terrorism-Related Disaster Event(s)

Improvised explosive devices (IEDs) and suicidal attempts on security forces and law enforcing agencies were the most common terrorism-related disasters experienced and reported by the nurses. The incidence of mass shooting at the Bethel Memorial Methodist Church in the city was also mentioned. Summarizing, all respondents had experienced, responded to and managed a terrorism-related disaster at the TC.

“*I was at routine work at the TC when a suicidal attack was reported. I also experienced the bomb blast in the heart of the city. God! (holding the head in grief) both unfortunate and tragic events resulted in multiple casualties.” (Nurse 2, Male)*

Another female nurse (Nurse 4) added that:

“*I still remember August 2016 where a suicide bombing killed more than 50 people (majority of them were lawyers). The injured list was separate. It was a horrifying condition for all of us.”*

#### Subtheme 1b: Disaster-Related Information Sources and Call-Up Mechanism

Imperatively speaking, as soon as a disaster occurs, healthcare practitioners should be informed promptly so they can respond efficiently and effectively. Therefore, a sound and responsive information management system plays a vital role across the hospitals as well as other affiliating institutes. This allows in reducing economic losses and mitigates the number of injuries or deaths that may result from a disaster. For that reason, the nurses were inquired about their information sources and call-up mechanism once a terrorism-related disaster has occurred.

“*We have our institutional/departmental WhatsApp groups where updated news and events are shared repeatedly. Additionally, social media and the National News Network is also a prompt source of disaster-related events. Besides, Quetta is a small city and once an unfortunate incidence occurs, the news is spread in no time.” (Nurse 1, Female)*

The respondents were also inquired about the call-up mechanism in case of disaster-related emergencies. All off-duty nurses of the TC receive a phone call from the hospital administration in case of a terrorism-related event. Moreover, it is acknowledged that they must report the TC as soon as possible once they have received the unfortunate news through any mean necessary. In short, the information sources and call-up mechanism adopted by the hospital in contacting nurses during an emergency was well-appreciated by the study respondents.

“*We (nurses of the TC) reside in the hospital premises that is within 5 minutes walking distance when compared to the TC. Normally we will receive a phone call in case of emergencies. Other than that, it is one of our job descriptions to rush and report at the TC as soon as possible. The reporting mechanism is simple, straightforward and effective.” (Nurse 8, Female)*

### Theme 2: Response Toward the Terrorism-Based Disaster Event

#### Subtheme 2a: Professional Response

Response includes events and activities that address the short- and long-term effects of a disaster. A prompt and effective response provides immediate support to maintain life improves health and helps in reducing the overall impact of the disaster. Consequently, it is important to understand the response of the healthcare professionals during the time of a disaster. Associating to the responses we received, nurses were confident in responding to such terrorism-related disasters in a professional and proficient manner. Based on their disaster management experience and the number of encounters faced, our respondents assured that they are always prepared to respond to an emergency in a timely and specialized manner.

“*As soon as we are informed about an emergency (or we know it by any means), we arrange and organize essential equipment and supplies at the TC. This includes preparation of the operation theaters, ward beds and availability of the medicine trolleys. We make sure that everything is prepared and ready before the arrival of the victims.” (Nurse 4, Female)*.

#### Subtheme 2b: Personal Response

While responding to a disaster, healthcare professionals are also exposed to trauma and may develop post-traumatic anxiety, stress, and depression. This development of adversative conditions can negatively affect their psychosocial well-being. Therefore, it is equally vital to understand the psychological response of healthcare professionals in a hostile condition. Inline to what is being described, nurses of the current study reported a positive response and strong determination while responding and managing victims that are brought to the TC. Also, our respondents stated that they are physically and mentally ready to face any unfortunate condition that may happen at any time.

“*Once the victims are brought to the TC, the circumstances and environment is beyond explanation. Therefore, we must support the healthcare professionals, victims and their attendees both professionally and generally. We have to overcome the stressful and panic conditions and for that we are always mentally and physically ready.” (Nurse 6, Female)*

#### Subtheme 2c: Inclusive Response

Although our respondents were prepared (both professionally and psychosocially) in dealing a terrorism-related disaster, certain deficiencies were also noted during the interviews. As in other developing nations, healthcare institutes of Pakistan are faced with a non-existence of disaster management response mechanism and system. Hence nurses' response to the emergencies is based on their past experiences and encounters. Terrorism-related disasters are not predictable by any means, and healthcare institutes can get additional benefit in dealing such disasters if a real-time supporting tool for disaster response is introduced and implemented.

“*I have read about the emergency response checklists and smart response systems. In my views, implementation of a system at the TC while responding to a disaster can help us to a great extent. Now we are handling the situations based on our experiences and no operating guideline is available.” (Nurse 9, Male)*

### Theme 3: Preparedness of Terrorism-Related Disaster Management

#### Subtheme 3a: Current Level of Knowledge and Familiarity of Terrorism-Related Disaster Management

Preparedness is related to the measures taken to prepare for and reduce the effects of disasters. It provides a platform that helps in designing procedures and eventually results in saving lives. Within this context, effective disaster management requires adequate knowledge of the events that is critical for risk reduction. Therefore, assessing disaster management knowledge among healthcare professionals is vital in addressing disaster-related issues. For that very reason, we inquired about the level of knowledge and familiarity of terrorism-related disaster management among our study respondents. Nurses of the current study expressed strong reservations regarding their knowledge and familiarity of terrorism-related disaster management. In continuation, all respondents agreed that there are no training opportunities, nor any seminars/workshops offered that can help in improving their understanding of terrorism-related disaster management.

“*It is my second year at the TC. Since then I have received no formal training regarding terrorism-related disaster management. There is no information module or written material that can help in improving our knowledge. What we are doing is purely based on our experience gained from handing terrorism-related emergencies.” (Nurse 11, Female)*

#### Subtheme 3b: Workforce, Infrastructure, and Supplies

Another important component while discussing disaster preparedness is the availability of a management framework. However, developing countries are faced with lack of management framework because of limited health budgets, shortage of healthcare professionals, and overburdened healthcare system. The same was explained by the respondents when they were asked about the availability of workforce and infrastructure at the TC. However, the respondents were satisfied with the medicines and supplies that are available at the TC.

“*We are facing shortage of workforce at the TC. Same goes to the space as it is also limited. In case of a mass disaster we utilize other sections of the hospital. We do not mind going for an extra stretch and to overload ourselves because saving lives is our priority. (Nurse 1, Male)*

While discussing about the medicine and supplies, Nurse 9 (female) explained that:

*The medicine and supply trolleys are well-prepared by the pharmacists. Normally it is enough to handle 80-90 victims prior to any terrorism-related event.”*

#### Subtheme 3c: Triage, Communication, and Coordination

Triage refers to the order of treatment during a mass disaster. An effectual triage needs an operative and active coordination and communication system that can identify the treatment priorities while managing a mass disaster. Unexpectedly, our responders were unaware of this term and had little or poor knowledge of order of treatment. This is because of their poor knowledge of disaster management (as discussed above) and lack of coordination among different institutes.

“*Order of treatment! (Confused). As soon as the victims arrive, we manage them according to their needs and severity. We do not have a protocol in determining the order of the patients and frankly we do not have this idea of triage.” (Nurse 6, Female)*

Some issues related to lack of coordination and communication was also reported by the respondents. Within the TC, the coordination and communication were satisfactory, however; nurses had some reservation toward other departments of the hospital.

“*In routine days (other than a disaster event) patients are also referred to TC from causality department. If we can good coordination and communication, it would be much better to manage the patients and to prepare in advance.” (Nurse 3, Female)*

### Theme 4: Barriers Toward Terrorism-Related Disaster Management

#### Subtheme 4a: Safety Concerns and Issues

Recurrence of the disaster as well as people' (attendees of the victims) hostile behavior during an emergency was mentioned as a key barrier toward terrorism-related disaster management. Our respondent had serious concerns and displayed major uncertainties as they had experience multiple encounters of the same kind. Such hostile behaviors and actions of the people placed the safety of the healthcare professionals at risk and resulted in the destruction of institutional assets.

“*Once a terrorism-related event occurs (especially suicidal attack), there are chances of another attack because people will rush to the TC, crowd will gather, so will be an easy target for the terrorists. At the same time, people start agitation and clamoring because of their loved ones is in critical condition. It is very hard to concentrate on job when you have the thought of another suicidal attack on mind and angry crowd here and there.” (Nurse 11, Male)*

#### Subtheme 4b: Lack of Disaster Management Content

Another barrier that was cited by the nurses was absence of subjects related to disaster management in the nursing curriculum as well as during the training period. Practically speaking, the respondents reported to have no idea of disaster management once they start nursing practice at the healthcare institutes. Therefore, the only choice is to follow the practice and to perform what is being practiced at the hospital.

“*There is nothing about disaster management in the nursing course. We are also not taught about disaster management at the hospital during our residency or regular practice. This is an important subject and we must know about it.” (Nurse 14, Female)*

#### Subtheme 4c: Lack of Drills and Hands-On Trainings

Lastly, absence of disaster drills and hands-on trainings were emphasized as a potential barrier toward disaster management. Openly, there is no concept of disaster drill at the institute nor the nurses have attended any hands-on training or workshop that was aimed to improve their disaster management skills. Drill and hands-on sessions are important as in addition to skill development, it improves the confidence that results in an increased efficiency and efficacy during emergencies and crisis.

“*The hospital has never arranged training(s) related to disaster management. Unlike other institutes, we never had a mock exercise or drill to improve our response and skills in course of an emergency. It is simple; continue doing what we are doing every day.” (Nurse 8, Male)*

### Theme 5: Suggestions and Recommendations

Summarizing the replies, all respondents were of the same opinion when they were asked about their suggestion while managing a disaster. Expansion of TC (human resource, space, and supplies), provision of line of instructions, plans and protocols to the employees, training sessions and exercises, and periodical revision and assessment to assure its readiness and preparedness of the TC were key recommendations of the study respondents.

“*We have long way to go. We need space, healthcare professionals and enough supplies. Besides, the administration must build our skills through continuous training sessions and assure time by time that we are ready to face a disaster in an efficient manner.” (Nurse 15, Male)*

## Discussion

Terrorism is never meant to kill as many people as possible. Terrorism-related events are planned to instill fear among people, dislocate social function, and perturb the general well-being of societies (44). Manifestations of terrorism-related events at a societal level result in community dysfunction that further reshapes as indiscriminate insecurity hence rupturing of the social fabric (45). While reconstructing infrastructure is relatively easy, the rejuvenation of societies and societal trust is often intricate and uncertain (46). In line with what is being discussed, healthcare system is not exempted from heightened concern when terrorism strikes at a community level. In the nutshell, terrorism impacts individuals, communities, and society on multiple levels and the consequences are devastating in both short and long term.

The current study was aimed to assess the response and preparedness of nurses toward terrorism-related disaster management. We believe that the interviews extracted enough information that was able to answer the questions that were established earlier. Shifting our concerns to terrorism-related events in Pakistan, majority of the terrorism-related activities are reported from the province of Baluchistan (47). For decades, Baluchistan is in continuous unrest and is facing tremendous economic and social harm because of these terrorism-related events. Since the 9/11, Baluchistan remained the worst victim of terrorist attacks in Pakistan as more than 1,000 individuals were killed and 1,570 were injured in 52 major terrorist attacks in the past 12 years (48). Even though a 33% reduction of terrorist-related events was reported in 2019, 145 people still lost their lives and 528 were injured in attacks including bombings, target killings, and landmine blasts (49). The upsurge of terrorism resulted in the establishment of the National Disaster Management Authority (NDMA) in 2005 and subsequently Provincial Disaster Management Authorities (PDMA) in all provinces. Since then, substantial progress has been made in advancing emergency planning and preparedness for terrorism-related events at national and provincial levels. Along with the efforts of NDMA and PDMA, healthcare system of the country has also played a crucial role when providing care and rehabilitation services to the victims. Nevertheless, with all the progress, the state of preparedness and assessment of professional response of healthcare system while dealing with a terrorism-related disaster is unknown. Keeping this limitation of information in mind, the current research aimed to ascertain how responsive and prepared the healthcare professionals (nurses) while managing a man-made disaster are.

Nurses of the current study identified financial constraints, lack of human resource, and deprived healthcare infrastructure as a major barrier while managing a terrorism-related disaster. Because of terrorism, Pakistan's economy has suffered a direct and indirect cost of almost US $126.79 billion (50). Local business and international trade are also adversely affected and resulted in increased inflation and loss of business market share. Furthermore, terrorism also increased government expenditure on security to maintain law and order in the country. This reallocation of government resources decreased expenditure on social sector development and decreased economic growth (50). Although the Government of Pakistan is winning the war against terrorism (51), the country is still facing economical and financial issues that are playing a major role in providing ample facilities to the healthcare system. Nevertheless, the development of a highly equipped TC, appointment of specialized healthcare professionals, and continuous monitoring of the hospitals do reveal the sincerity of efforts while responding and preparing for a terrorism-related event.

Nurses form the backbone of a healthcare system, especially when there is war, conflicts, and disasters (52). Nurses play important roles throughout the therapeutic cycle and are among the first professionals to provide care for people affected by a terrorist attack (53). Therefore, nurses in active war zones, emergencies and dealing with terrorism-related event needs professional training and unique skill set (54). This need is also evident from our study whereby the respondents agreed that without adequate training and protection, they are placing their health as well as the safety of the patients in jeopardy. Correlating the training and development, nursing education is of paramount importance while managing a disaster. Consequently, the nursing curriculum should be sufficiently rationalized with the updated knowledge to effectively respond to terrorism-related disasters (55) which is also reported by the respondents of the current study.

Disasters and other health emergencies such as suicidal attacks, road-side bombings, and other forms of IEDs require tangible plans for management. The International Council of Nurses (ICN) Disaster Management Continuum Model suggests four main components: mitigation, preparedness, response, and recovery (56). The main purpose of this model is to lessen the negative impact on lives and infrastructure, improve recovery, and construct community pliability to disasters (56). Parallel to the ICN, The International Nursing Coalition for Mass Casualty Education (INCMCE) has also developed competencies for all nurses, as well as online modules for meeting competencies while managing a disaster (57). Regrettably, such guidelines or protocol were not known to the respondents of the current study. Our discussion with the senior nurses revealed that as conventional education is provided in the classrooms, nurses are often unaware of such reported guidelines. Additionally, as the job description requires attending the patients' need, least attention is given by the nurses in inquiring new information. As discussed above, a thorough revision of the nursing curriculum and provision of continuous medical education is one way to improve the deficiency and to update the knowledge level of the nurses. It is evident that appropriate education and training of nurses for disasters is important for optimizing the safe functioning and minimizing emotional and psychological damage (18). Competency-based education provides an international infrastructure for nurses to learn about emergency preparedness and response which is seemed to be lacking in the current healthcare system of Pakistan.

Although our respondents agreed that disaster preparedness is highly needed to handle unpredictable calamitous situations effectively, lack of training to work efficiently in disaster management was reported. Our findings are supported by Pourvakhshoori and colleagues whereby the authors also reported poor training of Iranian nurses while managing a disaster (18). It is vital that nurses are aware of effective triage, emergency treatment, evacuation, and reintegrating the victims back to their routine. In addition, nurses should also be trained to take care of the amputations and undertake a life and limb-saving treatment. In the nutshell, basic knowledge and skill set to address terrorism-related emergencies or events is highly important (58). Unfortunately, nurses of the current study were unaware of triage, reintegration, and recuperation that clearly reflect the urgent need of training and development of nurses especially in the context of terrorism-based disaster management.

Healthcare system is an ever-evolving profession and nursing is not an exception. For that reason, regular curricular review and renewal is compulsory in nursing education (59). Continuing education assures continued competence (60) and leads the nurses to combat the chaotic situations. Our qualitative analysis clearly reflected that emergency management should be included in the regular training curriculum of nursing schools in Pakistan and is also backed by the World Health Organization.

## Conclusion

Nurses are an integral part of healthcare system and play a vital role in responding to and managing terrorism-related disasters. The skills and expertise needed to address a terrorism-related disaster are well-understood but are lacking for various reasons. Interpreting nurses' views through the extracted themes exposed review and adaption of the nursing curriculum specific for terrorism-related disaster management. Furthermore, continuous on-job training and education was also highlighted that will aid in responding and managing the disasters in an efficient manner. Lastly, in-house programs for nurses to learn more about terrorism-related disaster management are needed that will be helpful in preparing them for possible encounters in the future.

## Limitations

Qualitative research is not statistically representative as well as it is difficult to investigate causality. Furthermore, as we targeted nurses of the trauma center, recruiting nurses from other department may provide different results. This is achievable with a mixed methodological study in near future.

## Data Availability Statement

The original contributions presented in the study are included in the article/supplementary material, further inquiries can be directed to the corresponding author/s.

## Ethics Statement

The studies involving human participants were reviewed and approved by Institutional review board, Faculty of Pharmacy and Health Sciences. The patients/participants provided their written informed consent to participate in this study.

## Author Contributions

FK, AR, MS, and NB conducted the literature review and developed the interview protocol and the guide. FK conducted the interviews while RI and SR monitored the process as observers. SH and ZI analyzed and drafted the manuscript, which was subject to critical revision by NA and FS. The study was supervised by FS and NA. All authors read and approved the final manuscript. All authors contributed equally.

## Conflict of Interest

The authors declare that the research was conducted in the absence of any commercial or financial relationships that could be construed as a potential conflict of interest.
